# Augmentation of Recipient Adaptive Alloimmunity by Donor Passenger Lymphocytes within the Transplant

**DOI:** 10.1016/j.celrep.2016.04.009

**Published:** 2016-04-28

**Authors:** Ines G. Harper, Jason M. Ali, Simon J.F. Harper, Elizabeth Wlodek, Jawaher Alsughayyir, Margaret C. Negus, M. Saeed Qureshi, Reza Motalleb-Zadeh, Kourosh Saeb-Parsy, Eleanor M. Bolton, J. Andrew Bradley, Menna R. Clatworthy, Thomas M. Conlon, Gavin J. Pettigrew

**Affiliations:** 1 School of Clinical Medicine, University of Cambridge, Cambridge CB2 0QQ, UK

## Abstract

Chronic rejection of solid organ allografts remains the major cause of transplant failure. Donor-derived tissue-resident lymphocytes are transferred to the recipient during transplantation, but their impact on alloimmunity is unknown. Using mouse cardiac transplant models, we show that graft-versus-host recognition by passenger donor CD4 T cells markedly augments recipient cellular and humoral alloimmunity, resulting in more severe allograft vasculopathy and early graft failure. This augmentation is enhanced when donors were pre-sensitized to the recipient, is dependent upon avoidance of host NK cell recognition, and is partly due to provision of cognate help for allo-specific B cells from donor CD4 T cells recognizing B cell MHC class II in a peptide-degenerate manner. Passenger donor lymphocytes may therefore influence recipient alloimmune responses and represent a therapeutic target in solid organ transplantation.

## Highlights

• Donor CD4 T cells provide cognate, but peptide-degenerate, help to all host B cells
• Antibody specificity is determined by concurrent B cell receptor ligation
• Passenger donor CD4 T cells can therefore augment host alloantibody responses
• Host NK cell allorecognition is critical for preventing this augmentation

## Graphical Abstract

**Figure:**
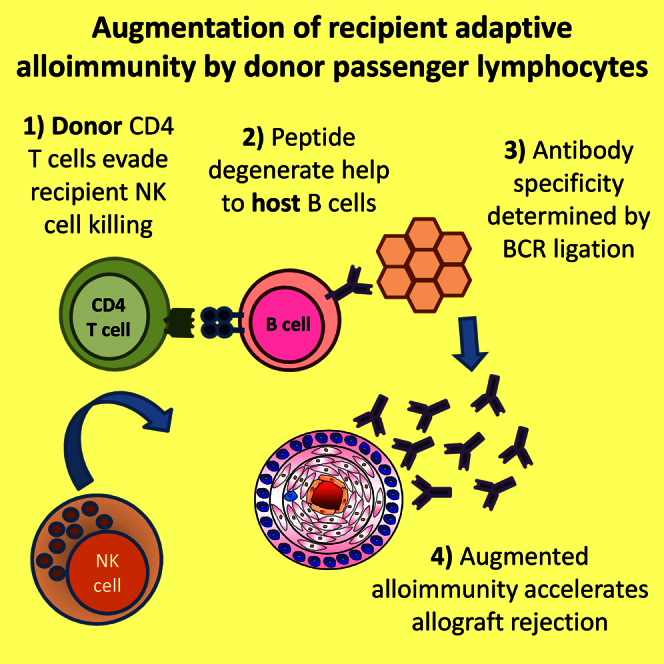

## Teaser

Transplanted organs may carry a significant number of tissue-resident lymphocytes, but their impact on transplant outcome is unknown. Harper et al. show that graft-versus-host recognition by passenger CD4 T cells within the allograft augments the subsequent host immune response to the allograft and hastens transplant rejection.

## Introduction

Solid organ transplantation provides an effective therapy for patients with kidney, liver, heart, and pulmonary failure. Long-term graft survival is limited by adaptive alloimmune responses directed against transplant (typically allogeneic major histocompatibility complex [MHC]) antigens, that are expressed within the organ and on endothelial cell surfaces and that interface with circulating recipient immune cells. In addition, it is appreciated that a substantial number of memory T cells reside within non-lymphoid tissues (Mueller et al., 2013, Shin and Iwasaki, 2013, Sathaliyawala et al., 2013). Solid organ allografts may therefore deliver “passenger” donor lymphocytes to the recipient after transplantation. Currently, little is known about whether passenger lymphocytes remain in the allograft or reach recipient secondary lymphoid organs or how long they survive, given that their likely recognition by natural killer (NK) cells might be expected to ensure rapid elimination. However, the precise role of NK cells in solid organ transplantation remains unclear (Gill, 2010, Hadad et al., 2014, van der Touw and Bromberg, 2010, Hidalgo et al., 2010), and early transplant studies indicate that circulating donor lymphocytes are often detectable in human transplant recipients, albeit in small numbers (Starzl et al., 1992a). Their presence may manifest as devastating, acute graft-versus-host (GVH) disease (Sharma et al., 2012), or as passenger lymphocyte syndrome, in which hemolysis is triggered by donor B cell recognition of mismatched ABO blood group antigens in the recipient (Nadarajah et al., 2013). Thus, the impact of passenger lymphocytes on the recipient immune response to the allograft has still to be clarified (Turner et al., 2014).

We have shown that in a murine heart transplant model with an isolated MHC class II-mismatch [B6(C)-H2-Ab1bm12/KhEgJ (bm12) to C57BL/6 (B6)], passenger bm12 CD4 T cell recognition of I-A^b^ MHC class II on host B cells triggers the production of anti-nuclear autoantibody, which causes allograft vasculopathy (Motallebzadeh et al., 2012, Win et al., 2009). GVH recognition by passenger lymphocytes may also contribute to graft rejection through other mechanisms. For example, activation of host dendritic cells (DCs) and macrophages following recognition of surface MHC class II by donor CD4 T cells could prompt more vigorous host alloimmunity from more effective processing and presentation of graft alloantigen as self-restricted peptide fragments.

To examine the possibility that passenger donor lymphocytes augment conventional host alloimmunity, we developed a murine transplant model incorporating a new bm12-derivative donor strain that expresses additional MHC class I and class II alloantigens to act as targets for conventional cellular and humoral allorecognition (Ali et al., 2016). Here we describe how in this model, heart allografts provoke autoantibody production in B6 recipients as a consequence of GVH recognition by passenger donor CD4 T cells. We show that even though donor CD4 T cells survive for only a few days after heart transplantation, their survival provokes a marked and long-lasting augmentation of cellular and humoral alloimmunity and results in early allograft rejection. However, this augmentation is prevented in completely mismatched strain combinations by rapid NK cell killing of donor lymphocytes. These data have important clinical implications, suggesting that partial MHC mismatch between donor and recipient to promote NK cells responses against passenger lymphocytes may reduce alloimmune responses.

## Results

### Heart Allografts with Isolated MHC Class I and Class II Disparities Provoke Allo- and Autoantibody Responses

**Figure 1:**
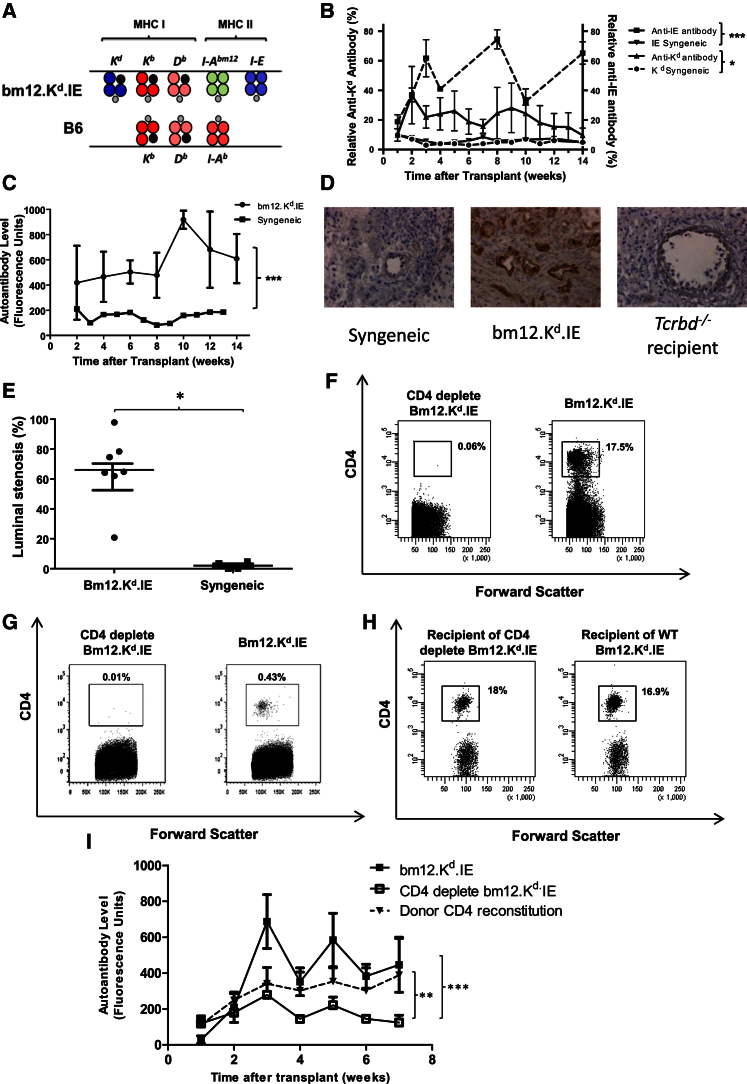
Heart Allografts with Isolated MHC Class I and Class II Disparities Provoke Alloantibody and Donor CD4 T Cell-Dependent Autoantibody Responses (A) A series of backcrosses were performed to generate the bm12.K^d^.IE donor strain that differs from B6 recipient mice at the I-A locus and mismatched H-2K^d^ and I-E loci. (B and C) In contrast to syngeneic heart transplants, bm12.K^d^.IE heart allografts triggered robust and durable IgG anti-K^d^ and (B) anti-I-E alloantibody and (C) anti-nuclear autoantibody responses. (D and E) Responses were associated with (D) complement C4d deposition on allograft endothelium, which was not observed in allografts transplanted into T cell-deficient *Tcrbd*^−/−^ recipients (scale bars, 100 μm), and (E) development of progressive allograft vasculopathy. (F and G) Treatment of donor bm12.K^d^.IE mice with anti-CD4 mAb resulted in depletion of CD4 T cells (F) in the circulation and (G) within the heart by the time of procurement of the heart graft 6 days later, as confirmed by flow cytometric analysis of PBMC and heart allograft homogenate. (H) The recipient splenic CD4 T cell population was unaltered by the donor treatment, indicating that antibody was not carried over to recipients. (I) Depleting the donor CD4 T cell compartment (CD4 deplete bm12.K^d^.IE) abrogated recipient IgG anti-nuclear autoantibody responses; these were restored by adoptively transferring purified donor CD4 T cells to recipients at the time of transplant. ^∗^p < 0.05, ^∗∗^p < 0.01, and ^∗∗∗^p < 0.001 (two-way ANOVA in B, C, and I; Mann-Whitney test in E). Data are representative of two independent experiments (B–H; mean and SEM of n = 7 mice per group in B, C, and E; n = 6 mice per group in F–H; or n = 4 mice per group in D) or one experiment (I; mean and SEM of n = 7 mice per group).

Human organs procured for transplantation, including kidney, liver, and heart, contain significant populations of effector and effector-memory CD4 and CD8 T lymphocytes (Figure S1). We therefore sought to examine the impact of these passenger donor lymphocytes on recipient adaptive alloimmune responses. To address this question, we developed a mouse strain that expressed multiple MHC alloantigens, sufficient to stimulate cellular and humoral alloimmunity, in addition to provoking humoral autoimmunity. A series of backcrosses were performed between bm12, B6.K^d^ (Honjo et al., 2004b), and B6.I-E (Conlon et al., 2012a) strains to derive the bm12.K^d^.IE strain, which differs from the B6 recipient strain at the classical MHC class I K and class II A and E loci (H-2^b^, K^bd^, A^bm12^, E, and D^b^; Figures 1A and S2).

When bm12.K^d^.IE hearts allografts were transplanted into B6 recipients, the additional MHC class I H-2K^d^ and class II I-E mismatched alloantigens provoked strong alloimmune responses, with production of long-lasting alloantibody to both antigens (Figure 1B). Recipients also developed anti-nuclear autoantibody (Figure 1C) that was comparable in magnitude to the responses previously observed in B6 recipients of bm12 heart allografts (Win et al., 2009, Motallebzadeh et al., 2012). These antibody responses were associated with C4d complement deposition on heart graft endothelium (Figure 1D), which was not evident in syngeneic heart transplants, suggesting a humoral component to the allograft vasculopathy that developed within allografts by day 100 (Figure 1E).

### GVH Allorecognition Provokes Recipient Humoral Autoimmunity

To determine whether, as in the bm12 to B6 model, autoantibody production in B6 recipients of bm12.K^d^.IE heart allografts was due to donor CD4 T cell allorecognition of recipient I-A^b^ MHC class II (Callaghan et al., 2012, Win et al., 2009), bm12.K^d^.IE donor mice were treated with depleting anti-CD4 monoclonal antibody (mAb) before sacrifice. This resulted in profound depletion of circulating and tissue resident CD4 T cell compartments by the time of heart allograft procurement (Figures 1F and 1G). Anti-CD4 antibody was not carried over to the recipient (Figure 1H); nevertheless, donor treatment with anti-CD4 mAb abrogated the recipient autoantibody response (Figure 1I), confirming that passenger CD4 T cells within the bm12.K^d^.IE donor heart are responsible for initiating recipient humoral autoimmunity.

Despite the development of humoral autoimmunity, no overt autoimmune disease was observed in kidney, liver, skin, or native heart in B6 recipients up to 100 days after transplantation with a bm12.K^d^.IE heart allograft (Figure S3).

### Augmentation of Conventional Alloimmunity by GVH Allorecognition

**Figure 2:**
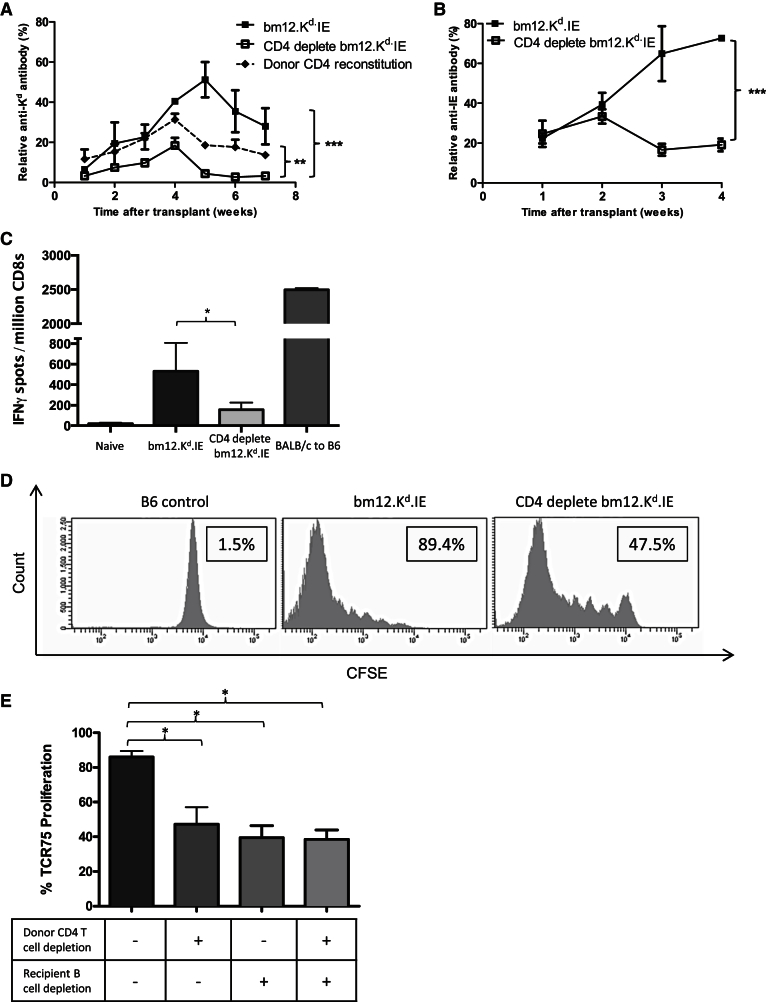
GVH Recognition by Passenger Donor CD4 T Cells within the Heart Allograft Augments Conventional Host Alloimmunity (A and B) Anti-K^d^ IgG alloantibody responses in B6 recipients of bm12.K^d^.IE heart allografts from CD4 T cell-depleted donors (CD4 deplete bm12.K^d^.IE) were significantly attenuated and restored by adoptive transfer of purified donor CD4 T cells to recipients at the time of transplant (A). Anti-I-E IgG responses were similarly abrogated (B). (C) Cytotoxic CD8 T cell alloresponses in B6 recipients of unmodified bm12.K^d^.IE heart allografts were weaker than those observed in B6 recipients of BALB/c heart grafts but significantly greater than those generated in recipients of CD4-T cell-depleted bm12.K^d^.IE heart allografts. (D and E) Indirect-pathway CD4 T cell responses, detected by quantifying proliferation of CFSE-labeled, K^d^-allopeptide-specific TCR75 CD4 T cells transferred 4 weeks after transplant, expressed as a percentage of parent population divided (boxes; D), were similarly reduced in B6 recipients of CD4 T cell-depleted bm12.K^d^.IE heart allografts (E). Donor CD4 T cell-mediated augmentation of host indirect-pathway CD4 T cell responses was not observed in B cell-depleted B6 recipients (E). ^∗^p < 0.05, ^∗∗^p < 0.01, and ^∗∗∗^p < 0.001 (two-way ANOVA in A and B; Mann-Whitney test in C and E). Data are representative of two independent experiments (A and B; mean and SEM of n = 6 mice per group) or one experiment (C–E; mean and SEM of n = 6 mice per group in C and E or n = 6 mice per group in D).

The ability, through specific targeting of the donor CD4 T cell population, to independently manipulate recipient autoimmune and alloimmune responses provided a means to examine whether GVH allorecognition augments host alloimmunity. Comparison of recipient cellular and humoral alloimmune responses in recipients of unmodified and CD4 T cell-depleted bm12.K^d^.IE heart allografts revealed that alloantibody responses against the H-2K^d^ alloantigen were substantially reduced in recipients of CD4 T cell-depleted allografts (Figure 2A and S4). Responses against the disparate donor MHC class II alloantigen were similarly ameliorated (Figure 2B). Allo- and autoantibody responses were restored in recipients of CD4 T cell-depleted bm12.K^d^.IE heart allografts by adoptive transfer of purified donor CD4 T cells at transplantation (Figures 1I and 2A).

The disparate H-2K^d^ alloantigen might be expected to act as a target for recognition by recipient cytotoxic CD8 T cells (Harper et al., 2015), but whereas B6 recipients of fully MHC-mismatched BALB/c heart allografts generated robust CD8 T cell responses, the response in recipients of unmodified bm12.K^d^.IE heart allografts was weak and transient (Figure 2C). Nevertheless, cytotoxic CD8 T cell responses were barely detectable in recipients of CD4 T cell-depleted bm12.K^d^.IE heart transplant recipients at any time point (Figure 2C). Helper CD4 T cell alloresponses were also examined in the recipient groups, by evaluating proliferation of TCR75 CD4 T cells that were adoptively transferred 5 weeks after the heart transplant. TCR75 CD4 T cells recognize K^d^ alloantigen via the indirect pathway (Ali et al., 2013) as self-I-A^b^-restricted, but not donor-I-A^bm12^-restricted, allopeptide (Honjo et al., 2004a, Conlon et al., 2012b). In recipients of CD4 T cell-replete heart grafts, marked TCR75 T cell proliferation was observed, indicating ongoing presentation of immunogenic K^d^ allopeptide epitope. In contrast, TCR75 T cell responses in recipients of CD4 T cell-depleted hearts were approximately 50% weaker (Figure 2D).

### GVH Allorecognition Contributes to Allograft Rejection

**Figure 3:**
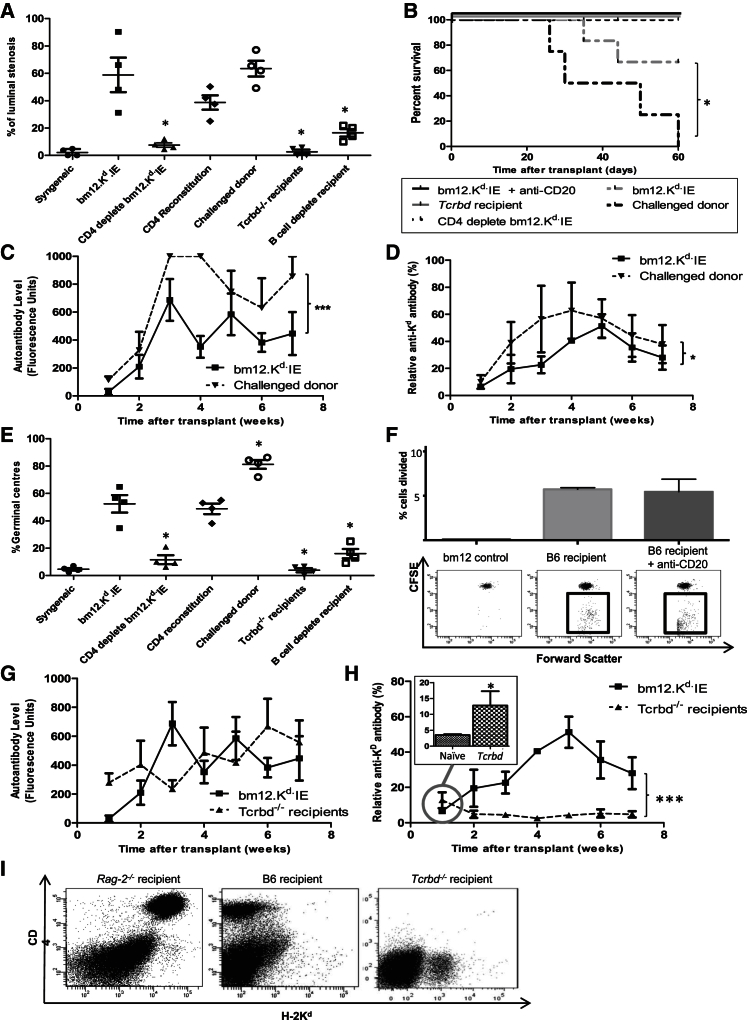
GVH Recognition by Passenger Donor CD4 T Cells Accelerates Heart Allograft Rejection but Is Dependent upon the Host T-B Cell Axis (A and B) Compared to B6 recipients of T cell-replete bm12.K^d^.IE heart allografts, allograft vasculopathy was significantly less severe in allografts from T cell-depleted donors (A), with all hearts beating strongly at harvest (B); vasculopathy was restored by adoptive transfer of purified donor CD4 T cells at transplant (CD4 reconstitution; A). (C and D) Transplantation of B6 recipients with heart allografts from bm12.K^d^.IE donors challenged with a B6 skin graft (challenged donor) provoked stronger (C) anti-nuclear IgG autoantibody and (D) anti-K^d^ IgG alloantibody responses, with heart grafts rejected more rapidly (B). (E) Allograft rejection is dependent upon host T and B cells (A), with the kinetics of rejection and the development of allograft vasculopathy in the different experimental groups mirroring recipient splenic GC activity. (F) Host B cells are not required for optimal GVH activation of donor CD4 T cells, because upon transfer of CFSE-labeled bm12.K^d^.IE CD4 T cells, the alloreactive fraction (boxed) divided similarly robustly in wild-type and B cell-depleted B6 recipients. (G and H) Compared to wild-type B6 recipients, transplantation of bm12.K^d^.IE allografts into T cell-deficient *Tcrbd*^−/−^ B6 recipients provoked similar anti-nuclear IgG autoantibody responses (G) but weak and transient anti-K^d^ IgG alloantibody with higher levels of alloantibody than observed in control naive serum achieved at week 1 (inset; H), without development of splenic GC activity (E). (I) Whereas adoptively transferred bm12.K^d^.IE CD4 T cells are readily detectable 7 days after transfer into *Rag-2*^−/−^ B6 mice, they were undetectable after transfer into wild-type B6 or *Tcrbd*^−/−^ mice. ^∗^p < 0.05, ^∗∗^p < 0.01, and ^∗∗∗^p < 0.001 (Mann-Whitney test in A, E, and H, inset [comparisons in A and E are to the bm12.K^d^.IE group]; log rank [Mantel-Cox] test in B; two-way ANOVA in C, D, G, and H). Data are representative of one experiment (A–F and I; mean and SEM of n = 4 mice per group in A–F or n = 3 mice per group in I) or two independent experiments (G and H; mean and SEM of n = 4 mice per group).

The marked reduction in the alloimmune response to CD4 T cell-depleted bm12.K^d^.IE heart allografts ameliorated graft rejection, in that vasculopathy was minimal in heart allografts from CD4 T cell-depleted donors and comparable to that observed in syngeneic heart transplants (Figure 3A). In addition, all heart transplants from CD4 T cell-depleted donors were beating strongly at day 50 (Figure 3B). Adoptive transfer of donor CD4 T cells at time of transplant to recipients of CD4 T cell-depleted bm12.K^d^.IE heart allografts restored the development of allograft vasculopathy (Figure 3A).

In contrast to human organs where memory T cell populations dominate (Figure S1), the CD4 T cell compartment in mice housed in specific-pathogen-free conditions is maintained in a largely naive state. We therefore sought to examine how memory CD4 T cells within an allograft might influence host alloimmunity, by priming Bm12.K^d^.IE donors with a B6 skin graft 6 weeks before procurement of the heart allograft to generate resident memory T cells. Heart allografts from such donors were rejected more rapidly by B6 recipients than were heart grafts from naive donors, and they triggered augmented auto- and alloantibody responses (Figures 3A–3D).

### Recipient T and B Cells Are Essential Mediators of the Accelerated Rejection Triggered by Early GVH Recognition

Amplification of the recipient alloreactive T-B lymphocyte axis is likely the principal mechanism by which donor CD4 T cell GVH recognition triggers accelerated graft rejection, because host germinal center (GC) alloantibody responses were less established in recipients of T cell-depleted, than T cell-replete, heart allografts (Figure 3E), as was complement C4d deposition on allograft endothelium (Figure 1D). Similarly, heart grafts were not rejected, and developed only minimal vasculopathy, when transplanted into either B cell-depleted (Figure S5) or T cell-deficient *Tcrbd*^−/−^ recipients (Figures 3A and 3B). To examine whether the augmentation in recipient CD4 T cell responses triggered by donor GVH recognition was dependent upon host B cell immunity, B cell-depleted recipients were transplanted with either CD4 T cell-replete or T cell-deficient bm12.K^d^.IE heart grafts, and proliferation of transferred TCR75 T cells was examined as earlier. For recipients of CD4 T cell-replete heart grafts, proliferation of adoptively transferred TCR75 T cells was substantially less in B cell-depleted than in untreated recipients (Figure 2E) and approximated that observed in untreated recipients of CD4 T cell-depleted bm12.K^d^.IE heart allografts. Furthermore, unlike B cell-replete recipients, proliferation of transferred TCR75 CD4 T cells in B cell-depleted recipients was not influenced by depletion of donor CD4 T cells (Figure 2E). The role of recipient B cells in GVH-mediated augmentation of recipient T cell alloreactivity does not simply reflect function as the major cell population expressing target I-A^b^ for optimal GVH activation of donor CD4 T cells, because the latter still divided readily in B cell-depleted donors (Figure 3F), such that no carboxyfluorescein succinimidyl ester (CFSE) staining was detectable in the sub-population of alloreactive bm12.K^d^.IE CD4 T cells as early as 3 days after transfer (Figure 3F).

**Figure 4:**
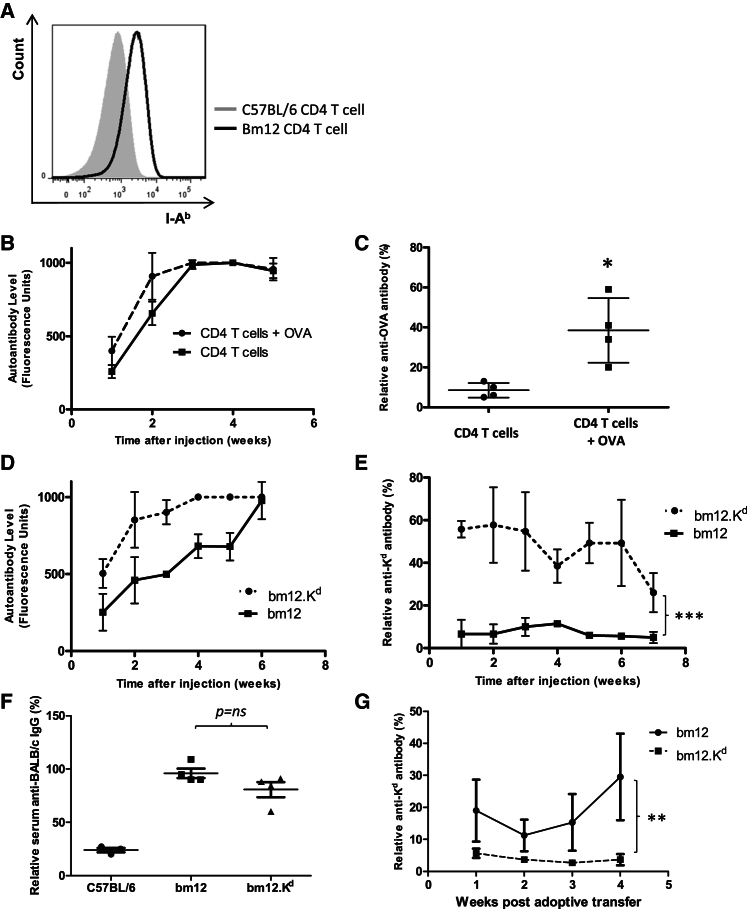
Peptide-Independent Recognition of the B Cell MHC Class II Complex by Donor CD4 T Cells Promotes Plasma Cell Differentiation but Requires Concurrent BCR Ligation (A) Seven days after transfer of bm12 CD4 T cells into B6 hosts, flow cytometric analysis of the splenic CD19^+ve^ B cell compartment demonstrates global upregulation of MHC class II expression, which is not evident upon transfer of syngeneic B6 CD4 T cells. (B and C) Whereas intravenous transfer of *Tcrbd*^−/−^ B6 mice with bm12 CD4 T cells provoked anti-nuclear IgG autoantibody (B), only those mice simultaneously immunized subcutaneously with ovalbumin (OVA) protein developed anti-OVA IgG (C). (D and E) Similarly, intravenous transfer of *Tcrbd*^−/−^ B6 mice with either purified bm12 CD4 T cells or bm12 CD4 T cells that expressed H-2K^d^ transgene (bm12.K^d^) provoked anti-nuclear IgG autoantibody (D), but anti-K^d^ IgG alloantibody was only generated in *Tcrbd*^−/−^ B6 mice that received bm12.K^d^ CD4 T cells (E). (F and G) Adoptive transfer of purified bm12.K^d^ CD4 T cells into T cell-deficient *Tcrbd*^−/−^ bm12 recipients of a BALB/c heart allograft confirmed not only that bm12 and bm12.K^d^ CD4 T cells can provide help for generating humoral alloimmunity, as determined by flow cytometric detection of bound test sera to target BALB/c BMDCs (F) but also that bm12.K^d^ CD4 T cells are tolerant of self (I-A^bm12^)-restricted H-2K^d^ peptide and do not provide help for generating anti-K^d^ IgG alloantibody (G). ^∗^p < 0.05, ^∗∗^p < 0.01, and ^∗∗∗^p < 0.001 (Mann-Whitney test in C and F; two-way ANOVA in E and G). Data are representative of three independent experiments (A; n = 6 mice per group) or one experiment (B–G; mean and SEM of n = 4 mice per group).

Although transplantation of bm12.K^d^.IE hearts into *Tcrbd*^−/−^ recipients prompted autoantibody and weak alloantibody responses (Figures 3G and 4H), there was no associated GC activity (Figure 3E). Thus, the requirement for host CD4 T cells in bm12.K^d^.IE heart graft rejection appears to reflect provision of essential help for development of sophisticated host humoral alloimmunity, a function not provided by donor CD4 T cells; transferred donor CD4 T cells are rapidly killed by adaptive alloimmune recognition in B6 hosts, because whereas bm12.K^d^.IE CD4 T cells were readily identified 7 days after transfer into *Rag*-2^−/−^ hosts, they were undetectable following transfer into wild-type B6 hosts (Figure 3I). Hence, it is unlikely that the donor CD4 T cells survive long enough to contribute directly to the progression of allograft vasculopathy. Their effect appears to be mediated principally through a relatively short-lived interaction with host B cells, but prolonged augmentation of humoral alloimmunity is dependent upon additional help from host CD4 T cells.

### Peptide-Independent Recognition of the B Cell MHC Class II Complex by Donor CD4 T Cells Promotes Plasma Cell Differentiation but Requires Concurrent B Cell Receptor Ligation

In considering how donor CD4 T cells amplify humoral alloimmunity, transfer of bm12 CD4 T cells into B6 hosts prompted upregulation of MHC class II expression on mature B cells (Figure 4A), in keeping with global activation from recognition of all allogeneic MHC class II complexes on their surface. Yet only a limited repertoire of antibody directed against nuclear self-antigen was produced (data not shown). To examine the hypothesis that plasma cell differentiation requires B cell receptor (BCR) ligation, in addition to cognate interaction between the MHC class II complex and the donor CD4 T cell, *Tcrbd*^−/−^ B6 mice were challenged with purified bm12 CD4 T cells and immunized with ovalbumin (OVA) protein. In this situation, CD4 T cell help for humoral responses can only be provided by the transferred donor CD4 T cells. Control *Tcrbd*^−/−^ mice received bm12 CD4 T cells only. As expected, mice in both groups developed anti-nuclear autoantibody, but anti-OVA immunoglobulin G (IgG) responses were only detectable in the group immunized with OVA (Figures 4B and 4C). Similarly, challenge of *Tcrbd*^−/−^ B6 mice with CD4 T cells from bm12 mice that expressed transgenic H-2K^d^ antigen (bm12.K^d^) provoked autoantibody, but also strong anti-K^d^ IgG alloantibody, which was not observed in *Tcrbd*^−/−^ B6 mice challenged with bm12 CD4 T cells (Figures 4D and 4E). Bm12.K^d^ CD4 T cells are selected against reactivity to self (I-A^bm12^)-restricted K^d^ peptide and are unable to provide help to K^d^-specific bm12 B cells for generating anti-H-2K^d^ antibody (Figures 4F and 4G). Thus, their provision of help for generating anti-H-2K^d^ antibody in B6 hosts reflects peptide-degenerate direct-pathway allorecognition of I-A^b^ MHC class II on H-2K^d^-specific B6 B cells that, with simultaneous BCR ligation, provokes class-switched alloantibody. These alloantibody responses presumably explain why bm12.K^d^.IE CD4 T cells are undetectable within a week of transfer into B6 *Tcrbd*^−/−^ mice but survive long term in *Rag-2*^−/−^ mice (Figure 3I). In summary, despite being tolerant of H-2K^d^ antigen on the bm12 background, bm12.K^d^ CD4 T cells provoke anti-K^d^ alloantibody when transferred into B6 hosts; this alloantibody results in rapid destruction of the bm12.K^d^ CD4 T cells.

### NK Cell Allorecognition Is Essential for Preventing GVH-Mediated Amplification of the Host Adaptive Alloimmune Response

**Figure 5:**
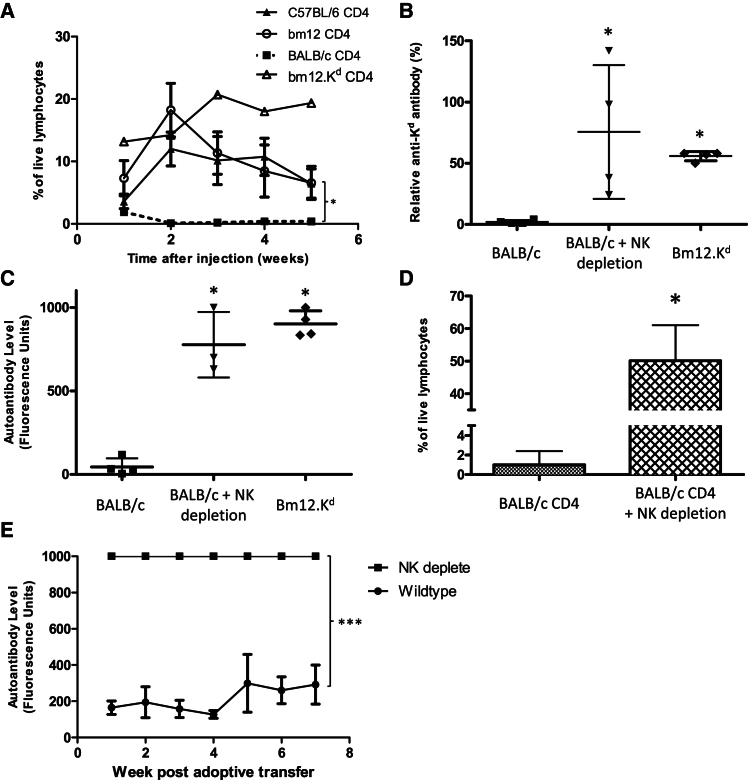
NK Cell Allorecognition Is Essential for Preventing GVH-Mediated Amplification of the Host Adaptive Alloimmune Response (A) Flow cytometric analysis of the peripheral blood mononuclear cell fraction demonstrates that whereas B6, bm12, and bm12.K^d^ CD4 T cells survive long-term following adoptive transfer into *Rag-2*^−/−^ mice, BALB/c CD4 T cells are rapidly undetectable. (B–D) Analysis of sera 4 weeks after transfer reveals that unlike transfer of bm12.K^d^ CD4 T cells, transfer of BALB/c CD4 T cells to *Tcrbd*^−/−^ B6 mice does not provoke (B) anti-K^d^ IgG alloantibody or (C) anti-nuclear IgG autoantibody. (D) In contrast, circulating BALB/c CD4 T cells are detectable 2 weeks after transfer into B6 *Rag-2*^−/−^ mice depleted of NK cells by administration of anti-NK1.1 mAb, and their transfer into NK cell-depleted *Tcrbd*^−/−^ B6 mice provokes (B) anti-K^d^ IgG alloantibody and (C) anti-nuclear autoantibody. (E) Anti-nuclear autoantibody responses are not generated by adoptive transfer of BALB/c CD4 T cells into wild-type B6 mice unless NK cells are first depleted. ^∗^p < 0.05, ^∗∗^p < 0.01, and ^∗∗∗^p < 0.001 (two-way ANOVA in A and E; Mann-Whitney test in B–D). Data are representative of two experiments (mean and SEM of n = 4 mice per experimental group).

Whether the amplification of host humoral immunity by GVH recognition is an intrinsic component of the alloresponse or is dependent upon the degree of MHC mismatch between donor and recipient has not been addressed. Given that donor bm12.K^d^.IE CD4 T cells survive long term in *Rag-2*^−/−^ B6 hosts (Figure 3I), we examined whether innate immune evasion, and specifically lack of NK cell allorecognition of donor lymphocytes, was critical in triggering autoantibody generation. In this regard, CD4 T cells purified from the completely mismatched BALB/c donor strain did not survive when injected into B6 *Rag-2*^−/−^ hosts and did not provoke humoral auto- or alloimmunity upon injection into B6 *Tcrbd*^−/−^ mice (Figures 5A–5C). This contrasts with long-term survival and development of strong IgG allo- and autoantibody when purified CD4 T cells from the less mismatched strains were injected (Figures 5A–5C). Furthermore, depletion of NK cells by administration of anti-NK1.1 antibody, in the B6 *Rag-2*^−/−^ recipients, resulted in long-term survival of transferred BALB/c CD4 T cells and, in *Tcrbd*^−/−^ recipients, provoked class-switched auto- and alloantibody responses (Figures 5B–5D) that were even stronger than those observed upon administration of CD4 T cells from the less mismatched donor strains. NK T cells, which also express NK1.1, do not develop in *Tcrbd*^−/−^ mice (Figure S6); these experiments therefore serve as an apposite control that the administered anti-NK1.1 antibody is acting principally upon NK cells. Irrespective of GVH recognition, injection of BALB/c CD4 T cells into wild-type, immunocompetent B6 mice would be expected to provoke alloantibody, but autoantibody was only produced if host NK cells were depleted simultaneously (Figure 5E), confirming that elimination of the transferred donor CD4 T cell population, by either host cytotoxic CD8 T cell or alloantibody responses, does not occur quickly enough to obviate a GVH response and that NK cell allorecognition is instead essential for its prevention.

### Host NK Cell Allorecognition Prevents Donor Passenger Lymphocytes from Triggering Accelerated Rejection of Completely MHC-Mismatched Allografts

**Figure 6:**
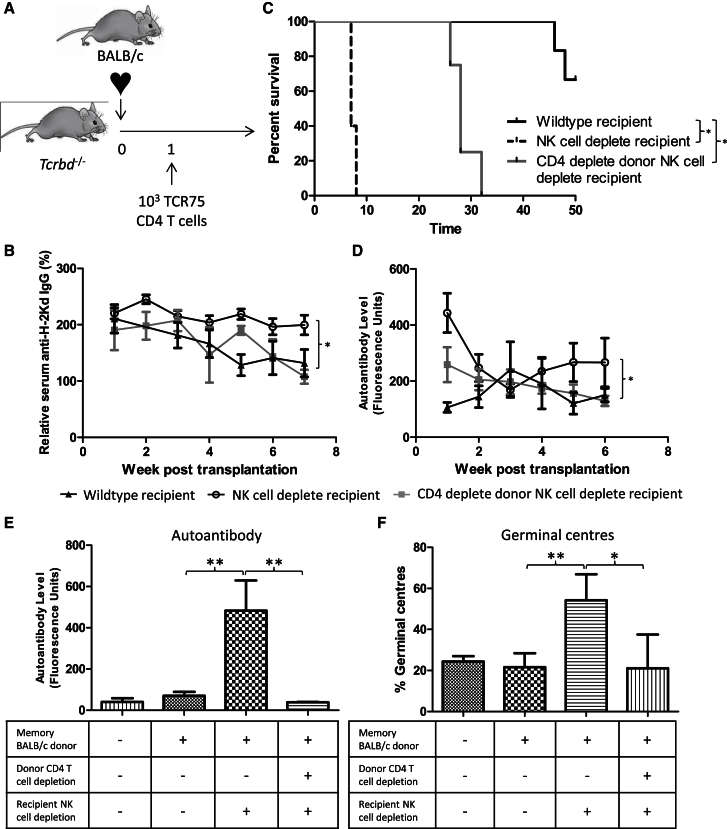
Host NK Cell Alloreactivity Is Critical for Preventing Donor Passenger Lymphocytes from Augmenting Host Adaptive Alloimmunity (A) A model of alloantibody-mediated allograft vasculopathy was developed in which 10^3^ TCR75 CD4 T cells are transferred into B6 *Tcrbd*^−/−^ mice at transplant with a BALB/c heart allograft. (B–D) In contrast to reconstituted *Tcrbd*^−/−^ (CD4^+ve^*Tcrbd*^−/−^) recipients, depletion of NK cells in the reconstituted *Tcrbd*^−/−^ recipients (NK cell^−ve^ host) results in (B) more rapid allograft rejection, (C) stronger anti-K^d^ IgG alloantibody responses, and (D) generation of anti-nuclear autoantibody. The impacts of recipient NK cell depletion were ameliorated by depleting CD4 T cells in donor mice (CD4^−ve^ donor, NK cell^−ve^ host) before heart allograft procurement. Analysis of allograft vasculopathy was not performed due to the rapid rejection of the NK cell-depleted recipient group. (E and F) Acute rejection of BALB/c heart allografts was prevented by administration of anti-CD154 mAb at transplant to B6 recipients. (E) Anti-nuclear IgG autoantibody responses are shown 5 weeks after transplant in recipients of heart allografts from unmodified BALB/c donors or from either BALB/c donors challenged with a B6 heart allograft 6 weeks earlier or similarly primed BALB/c donors that were depleted of CD4 T cells before heart allograft procurement. An additional group of recipients of allografts from primed donors were depleted of NK cells at transplant. (F) Corresponding splenic GC activity in the preceding recipient groups 5 weeks after transplantation. ^∗^p < 0.05, ^∗∗^p < 0.01, and ^∗∗∗^p < 0.001 (log rank [Mantel-Cox] test in two-way ANOVA in B and C). Data are representative of two independent experiments (B–D; mean and SEM of n = 5 mice per group B and D or n = 5 mice per group C).

These observations suggest that GVH-mediated amplification of host alloimmune effector responses is normally prevented in MHC-mismatched transplant models by host NK cell alloreactivity. The role of NK cells in rejection of completely mismatched BALB/c hearts by B6 recipients was therefore examined. However, in this model of acute rejection, unmodified B6 recipients reject BALB/c heart allografts within days, and it seemed unlikely that host NK cell depletion would influence such a robust rejection response. Instead, a further model of chronic alloantibody-mediated allograft vasculopathy was developed in which B6 *Tcrbd*^−/−^ recipients of BALB/c heart allografts are reconstituted at transplantation with B6 TCR75 CD4 T cells but at limiting numbers (10^3^ per mouse), such that rejection occurs slowly and is mediated by anti-H-2K^d^ GC alloantibody responses, with help provided by differentiation of the transferred TCR75 T cells to follicular helper T cells (Figure 6A). In contrast to the gradually evolving anti-K^d^ alloantibody responses observed in NK cell-replete recipients, responses in the NK cell-depleted recipients were stronger (Figure 6B), and the heart grafts were rejected within the first week (Figure 6C). Autoantibody was also generated in the NK cell-depleted recipients (Figure 6D), confirming the development of GVH responses mediated by donor BALB/c CD4 T cells. Critically, autoantibody generation, the augmented alloantibody response, and rapid allograft rejection were ameliorated in NK cell-depleted recipients by depletion of CD4 T cells from the BALB/c donor before heart graft procurement (Figures 6B–6D).

Finally, to test the relevance of our findings to a model in which graft rejection is prevented by administration of immunosuppression, as occurs routinely in clinical practice, heart allografts from BALB/c donor mice that had been challenged 6 weeks earlier with a B6 skin graft were transplanted into B6 recipients that were treated with anti-CD154 monoclonal antibody at transplantation. In B6 recipients of heart grafts from unmodified donors, this protocol results in long-term allograft survival (Larsen et al., 1996, Ali et al., 2016), without development of autoantibody (Figure 6E), but in recipients of heart allografts from challenged donors (that contained memory passenger CD4 T cells), depletion of NK cells at transplantation resulted in development of anti-nuclear autoantibody and more pronounced splenic GC activity (Figures 6E and 6F). Despite the robust GC response, anti H-2K^d^ alloantibody responses were not observed (data not shown). Depletion of CD4 T cells in the donor before heart allograft procurement abrogated the autoantibody response (Figures 6E and 6F).

## Discussion

Although the presence of donor lymphocytes in the circulation of recipients of solid organ allografts was first demonstrated more than 2 decades ago (Starzl et al., 1992a, Starzl et al., 1992b), the extent to which they affect recipient alloimmunity has remained unclear. Clarification of the contribution of passenger donor lymphocytes to graft rejection has become more pertinent with the realization that non-lymphoid tissue contains substantial populations of either resident or circulating memory T lymphocytes, and their presence has been described within all solid organs currently transplanted in humans (Casey et al., 2012, Sathaliyawala et al., 2013). Here, we used a combination of donor CD4 T cell depletion and adoptive transfer of donor CD4 T cells, in conjunction with transplantation of heart allografts from primed donors, to demonstrate that GVH allorecognition by donor CD4 T cells augments recipient alloimmunity and that this augmentation is more pronounced for allografts procured from donors sensitized against recipient MHC. Our findings thus reveal a mechanism by which donor lymphocytes may influence graft rejection and suggest that their impact may be more important than previously considered.

**Figure 7:**
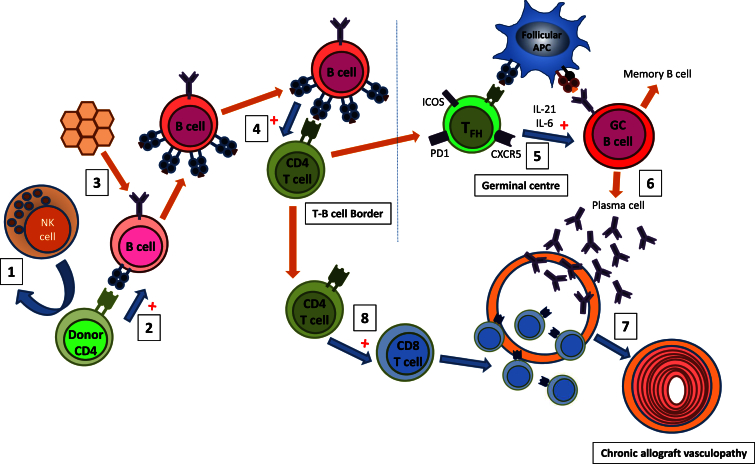
Proposed Model for Augmentation of Host Adaptive Alloimmunity by Passenger Lymphocytes NK cell allorecognition normally results in rapid destruction of donor passenger lymphocytes within solid organ allografts (1). If NK cell allorecognition is avoided, peptide-degenerate GVH recognition by donor CD4 T cells can activate all recipient B cells (2), but class-switched antibody secretion is dependent upon simultaneous ligation of BCR with target antigen (3). Activated B cells drive enhanced activation of host CD4 T cells with indirect allospecificity (4), which provide reciprocal help for development of GC alloantibody responses (5), presumably reflecting unique T follicular helper cell function of host CD4 T cells in providing cognate, allopeptide-specific help (5). This results in long-term augmentation of humoral alloimmunity (6), with more rapid progression of allograft vasculopathy and early allograft failure (7). Enhanced activation of indirect-pathway CD4 T cells may also contribute to allograft rejection through the provision of help for generating heightened host CD8 T cell cytotoxic alloresponses (8).

Pivotal to this augmentation of host alloimmunity is the ability of donor CD4 T cells to recognize host MHC class II via the direct pathway (Ali et al., 2013). This provides an unusual form of peptide-degenerate help, reflecting the unique nature of direct-pathway allorecognition (Macdonald et al., 2009, Ali et al., 2013), in which the precursor frequency of CD4 T cells that respond to a particular MHC class II alloantigen is 100- to 1,000-fold greater than for the response against conventional, self-restricted peptide antigen, because all MHC class II alloantigen complexes are recognized as foreign, irrespective of bound peptide. This results in activation of all recipient B cells, but we detail that differentiation to an IgG antibody-secreting plasma cell is dependent upon simultaneous B cell receptor ligation. Thus, although donor CD4 T cells can provide help to recipient B cells in an antigen-independent fashion, antigen specificity is maintained through the requirement for B cell receptor ligation. This atypical help does not, however, completely replicate conventional cognate help provided by CD4 T cells with self-restricted specificity for peptide derived from target antigen, because although GVH recognition by donor CD4 T cells could trigger auto- and alloantibody responses, these were not sustained in the absence of a recipient CD4 T cell population, and allograft rejection did not occur. Our findings thus reveal an interaction between donor and recipient T and B lymphocytes, as depicted in Figure 7.

Passenger lymphocytes only augmented recipient alloimmunity if they were not eliminated rapidly by recipient NK cells. The contribution of NK cell alloresponses to allograft rejection is still debated (Gill, 2010, Hadad et al., 2014, van der Touw and Bromberg, 2010). It has been suggested that host NK cells promote allograft rejection (Maier et al., 2001, Uehara et al., 2005, Kroemer et al., 2008) either through the destruction of opsonized donor cells or perhaps through regulation of T cell immunity (Maier et al., 2001). Our results suggest the converse: that a major function of NK cells is inhibition of destructive cellular and humoral alloimmunity that is triggered by passenger CD4 T cell GVH recognition. This accords with several studies reporting a key role for NK cells in allograft tolerance (Beilke et al., 2005, Yu et al., 2006). The mechanisms by which NK cells promote tolerance in these studies have not been firmly established, but inhibition of recipient alloimmune responses through killing of donor DCs may be important (Yu et al., 2006); in support, Laffont et al. (2008) have reported that NK cell-mediated destruction of donor DCs downregulates CD4 T cell alloimmunity. The different mechanism highlighted by our study—the killing of passenger lymphocytes—may be more clinically relevant, because irrespective of NK cell allorecognition, adaptive alloimmune recognition would be expected to result in prompt destruction of donor DCs; in the Laffont et al. (2008) study, CD8α^−/−^ mice were studied to obviate rapid killing by cytotoxic T lymphocytes. Our results reveal that the critical window for passenger donor CD4 T cells to augment host alloimmunity is within the first few days after transplant and that evasion of NK cell-mediated killing is essential for this effect. Donor CD4 T cells prompt host adaptive responses that engender their own rapid destruction, but the delay in development of these responses, at most a few days when compared to NK cell recognition, is sufficient for GVH recognition to occur.

What are the implications of our study for clinical solid organ transplantation? One could argue, on the basis of the derived nature of the bm12.Kd.IE donor strain and the lack of requirement for administration of immunosuppression, that the clinical relevance is limited. Similarly, it is perhaps surprising that passenger donor lymphocytes were present in sufficiently large numbers within heart allografts to provoke such a marked augmentation in the host’s alloimmune response. Against this, CD4 T cells could be readily detected in all sampled human organs that have been procured for transplantation but not used. In addition, to counter concerns regarding the wider applicability of the bm12.Kd.IE model, we employed an additional model of chronic allograft vasculopathy using completely mismatched BALB/c donor and B6 recipient strains. This model enabled clarification of the crucial role of host NK cells in killing donor hematopoietic cells; nevertheless, the potential for passenger donor lymphocytes to augment host alloimmunity was again observed. We further demonstrated in this model that memory donor CD4 T cells (as would be expected to be present within human allografts) were able to provide co-stimulation-independent help to naive recipient B cells for production of a GC autoantibody response.

With regards the seemingly large numbers of donor lymphocytes contained within our murine heart allografts, our experiments were not able to distinguish whether these were truly resident within the parenchyma or trapped within the microcirculation of the heart allograft, and it is possible that different procurement and storage techniques used in clinical transplantation denude a heart allograft of most of its passenger lymphocyte populations. Against this, deliberate flushing of our mouse hearts via the coronary arteries at explant (as typically occurs in clinical heart allograft procurement) did not make any appreciable difference to the numbers of CD4 T cells subsequently found within the heart allograft (data not shown). In any event, we stress that the heart allograft model was used as a means of delineating the precise mechanism by which donor CD4 T cells influence the host’s response to an allograft. In this respect, whereas the impact of passenger lymphocytes in clinical cardiac transplantation may be limited, lung, small bowel, and composite tissue allografts will almost certainly transfer large numbers of donor lymphocytes that originate from organized lymphoid tissue contained within the allograft. Transplant outcomes for these organs are poorer than for other organs; for example, a report of chronic face allograft rejection described the development of autoimmune, scleroderma-type features consistent with skin manifestations of chronic GVH (Petruzzo et al., 2015). One might therefore predict that transplant outcomes would be particularly poor for individuals that receive such organs from donors matched for killer cell immunoglobulin-like receptor (KIR) recognition (which occurs in approximately 50% of kidney transplant pairings; van Bergen et al., 2011), because the avoidance of immediate host NK cell detection would enable passenger donor lymphocytes to potentiate host alloimmunity. However, the impact of NK cell alloreactivity in transplant outcomes remains uncertain (Tran et al., 2013, van Bergen et al., 2011), possibly because studies to date have avoided the confounding impact of human leukocyte antigen (HLA) mismatches on allograft survival by including only HLA-matched donor-recipient combinations, whereas our findings suggest that KIR-ligand matching would compromise transplant outcomes when donor and recipient are mismatched additionally at the HLA class II loci.

It is likely that the ability of donor CD4 T cells to provide peptide-independent help to host B cells has implications beyond solid organ transplantation. In hematopoietic stem cell transplantation, an association between chronic GVH disease and humoral immunity is increasingly recognized (Nakasone et al., 2015, Dubovsky et al., 2014, Shimabukuro-Vornhagen et al., 2009, Svegliati et al., 2007). Our findings suggest that this may relate to a chimeric state in which the co-existence of populations of donor and recipient T and B lymphocytes tends to provoke destructive alloantibody responses. In addition, persistence of a mixed chimeric state implies that reciprocal NK cell tolerance to donor and host had been achieved (Narni-Mancinelli et al., 2013), which may be particularly relevant to strategies for hematological malignancy that use less toxic, non-myeloablative conditioning to initially establish mixed hematopoietic chimerism and then later convert to full donor chimerism by infusion of donor lymphocytes (Chang and Huang, 2013). Our findings suggest that inhibition of host NK cell alloresponses may enable GVH recognition by CD4 T cells within the subsequent donor infusion to provide promiscuous help for antibody production from residual host B cells that are concurrently engaging target antigen. This may explain reports documenting the development of humoral immunity against tumor antigen following establishment of mixed hematopoietic chimerism (Bellucci et al., 2004, Kremer et al., 2014). Similarly, the presence of donor CD4 T cells within donor lymphocyte infusions has been associated with loss of donor mixed hematopoietic chimerism (Kim et al., 2004, Hock et al., 2014), but rather than this being a bystander consequence of the general inflammatory milieu created by the GVH response (Hock et al., 2014), our results suggest that the loss may instead be due to cognate recognition of MHC class II on the surface of recipient alloreactive B cells by donor CD4 T cells.

In summary, we demonstrate an unexpected role for donor passenger CD4 T cells within allografts in the provision of help to recipient B cells for generating humoral responses directed against the transplant. Passenger donor lymphocytes may therefore influence recipient alloimmune responses more profoundly than previously considered and represent a therapeutic target in solid organ transplantation.

## Experimental Procedures

### Animals

B6 (H-2^b^) and BALB/c mice (H-2^d^) were purchased from Charles River Laboratories. Bm12 mice and T cell receptor-deficient mice (H-2^b^, *Tcrbd*^−/−^) B6.129P2-*Tcrb*^*tm1Mom*^*Tcrd*^*tm1Mom*^/J were purchased from The Jackson Laboratory. *Tcrbd*^−/−^ mice were backcrossed onto bm12 to create *Tcrbd*^−/−^.bm12 mice. B6 *Rag-2*^−/−^ mice (H-2^b^) were gifted by Prof. T. Rabbitts (Laboratory of Molecular Biology). TCR-transgenic *Rag-1*^−/−^ TCR75 mice (H-2^b^), specific for I-A^b^-restricted H-2K^d^_54–68_ peptide (Honjo et al., 2004a) and B6-Tg(K^d^)RPb (B6.K^d^) mice, which express the full sequence of H-2K^d^ (Honjo et al., 2004b), were gifted by Prof. P. Bucy (University of Alabama). B6.K^d^ mice were backcrossed onto a bm12 background to create bm12.K^d^ mice. B6 mice that lack I-A^b^ but express I-Eα (B6.I-E; Conlon et al., 2012a) were gifted by Prof. C. Benoist (Joslin Diabetes Center). The F_1_ offspring of Bm12.K^d^ and B6.I-E mice were bm12.K^d^.IE. All animals were maintained in specific pathogen-free facilities, and experiments were approved by the UK Home Office Animal (Scientific Procedures) Act of 1986.

### Heterotopic Cardiac Transplantation

Fully vascularized cardiac allografts were transplanted intra-abdominally (Conlon et al., 2012b). Rejection, defined as cessation of palpable myocardial contraction, was confirmed at explant. Grafts were excised at predetermined time points after transplantation and stored at −80°C or fixed in 10% buffered formalin. In certain experiments, heart allografts were retrieved from donor mice challenged with a recipient strain skin allograft 6 weeks earlier, or recipients were additionally injected intraperitoneally (i.p.) with 500 μg anti-CD154 mAb (clone MR-1; BE0017-1; Bio X Cell) on days −2 and 0 in relation to transplantation, a protocol that prevents acute allograft rejection but that results in development of chronic allograft vasculopathy.

### Dendritic Cell Purification and Culture

Bone marrow-derived dendritic cells (BMDCs) were prepared as described previously (Curry et al., 2007). Briefly, bone marrow (BM) was flushed from femurs and tibias with Hank’s balanced salt solution (Invitrogen). Cells were disaggregated by passing through a 40-μm mesh, and BM cells cultured in six-well plates at 3 × 10^6^/ml in 6-ml complete medium (RPMI 1640, 10% fetal calf serum [FCS], 100 IU/ml penicillin, 100 μg/ml streptomycin, and 2 mM L-glutamine; Invitrogen), supplemented with murine granulocyte-macrophage colony-stimulating factor (PeproTech) at 20 ng/ml and recombinant murine interleukin-4 (PeproTech) at 10 ng/ml. Cells were maintained by replacing half the culture medium with fresh medium on alternate days. Nonadherent cells were discarded on day 4, and DCs were used on day 8 for flow cytometric analysis.

### Assessment of Recipient Humoral Immunity

#### Autoantibody Quantification

Anti-nuclear autoantibody responses were determined by HEp-2 indirect immunofluorescence (The Binding Site), as described previously (Callaghan et al., 2012), by incubating test sera on slides coated with HEp-2 cells and detecting bound antibody with fluorescein isothiocyanate (FITC)-conjugated goat anti-mouse IgG (STAR 70; Serotec). For each test serum, photomicrographs were taken, and the intensity of staining was determined by integrated morphometric analysis using MetaMorph software. The fluorescence value was then derived by comparison with a standard curve, obtained for each assay by serial dilutions of a pooled hyperimmune serum that was assigned an arbitrary value of 1,000 fluorescence units.

#### Assay of Circulating Anti-MHC Class II I-E and Anti-BALB/c Alloantibody

Sera were collected from experimental animals weekly and analyzed for anti-I-E alloantibody (at week 4 in the case of BALB/c alloantibody) by flow cytometric detection of binding to target cells. Briefly, target B6.I-E and BALB/c BMDCs were first blocked with anti-mouse CD16/CD32 (clone 2.4G2; BD Pharmingen) and then incubated with serial dilutions (3-fold) of heat-inactivated test serum for 30 min. Bound alloantibody was detected with FITC-conjugated goat anti-mouse IgG (STAR 70; Serotec), and cells were analyzed by flow cytometry. For each sample, the geometric mean-channel fluorescence was obtained and plotted against dilution, and the area under the curve (AUC) was then calculated as a percentage of the AUC of a standard of pooled hyperimmune sera.

#### Determining Circulating Anti-H-2K^d^ Alloantibody and Anti-ovalbumin Antibody

Serum samples were collected from experimental animals weekly and analyzed for the presence of anti-H-2K^d^ IgG alloantibody by ELISA. In brief, 96-well ELISA plates (Immulon 4HBX; Thermo Scientific) were coated with recombinant conformational H-2K^d^ at 5 μg/ml in Na_2_CO_3_-NaHCO_3_ buffer (pH 9.6). Plates were blocked with 1% Marvel dried skimmed milk powder (Premier International Foods), tripling serial dilutions of test sera added and bound IgG antibody detected by incubating with biotinylated rabbit F(ab′)_2_ anti-mouse IgG (STAR11B; AbD Serotec) and ExtrAvidin Peroxidase conjugate (Sigma). Sure Blue substrate (KPL) was then added, the reaction was stopped by the addition of 0.2 M H_2_SO_4_, and the absorbance (optical density 450) was measured in a FluoStar Optima plate reader (BMG Labtech). For each sample, an absorbance versus dilution curve was plotted, and the AUC was calculated (Conlon et al., 2012a). The AUC of an experimental sample was expressed as the percentage of positive control (pooled hyperimmune) serum.

In certain experiments, mice were additionally immunized with OVA protein 100 μg in incomplete freund’s adjuvant subcutaneously. Anti-OVA antibody was assayed in a similar fashion, and performed on test sera 4 weeks after immunization, using an OVA-specific ELISA.

### CD8 T Cell IFN-γ Enzyme-Linked Immunospot

CD8 T cell enzyme-linked immunospot was performed as described (Sivaganesh et al., 2013). Briefly, purified CD8 T cells were mixed with irradiated BALB/c stimulator splenocytes and added to Multiscreen HTS filtration system plates (Millipore) that had been coated with anti-mouse interferon-γ (IFN-γ; BD Pharmingen) in 0.1 M bicarbonate buffer (pH 9.6). Plates were incubated at 37°C and 5% CO_2_ for 20 hr, and after washing, spots were developed with biotinylated rat anti-mouse IFN-γ (BD Pharmingen), followed by streptavidin-horseradish peroxidase and the substrate, H_2_O_2_, together with the 3-amino-9-ethylcarbazole color indicator. Plates were read (Autoimmun Diagnostika), and data were expressed as spot counts per 10^6^ responder CD8 T cells for each well.

### Flow Cytometry

Antigen-presenting cell-conjugated anti-mouse CD4 (RM4-5), FITC-conjugated anti-mouse CD19 (1D3), R-phycoerythrin (PE)-conjugated anti-mouse CD90.1/Thy1.1 (clone OX-7), PE-Cy7-conjugated anti-mouse CD4 (clone L3T4), PE-conjugated anti-mouse H-2K^d^ (SF1-1.1), and FITC-conjugated anti-mouse I-A^b^ (clone AF6-120.11) were purchased from BD Pharmingen. Peripheral blood (depleted of erythrocytes by incubating with 0.17 M NH_4_Cl red cell lysis buffer) and splenic single-cell suspensions were blocked with anti-mouse CD16/CD32 (clone 2.4G2; BD Pharmingen), before staining with the relevant antibodies and dead cell exclusion dye 7-aminoactinomycin D (BD Pharmingen). All cells were analyzed on a FACSCanto II flow cytometer with FACSDiva software (BD Biosciences).

### T Cell Proliferation Assay

Single-cell suspensions of splenocytes obtained from TCR75 mice were stained with 5 μM CFSE (Molecular Probes) in the dark for 5 min and then quenched with 5% FCS/PBS. CFSE-stained splenocytes (2 × 10^6^ to 5 × 10^6^) were injected intravenously (i.v.) into recipient mice and spleens harvested 4 or 7 days later; flow cytometry was performed using allophycocyanin-conjugated anti-CD4 plus PE-conjugated anti-CD90.1/Thy1.1 to identify TCR75 T cells. Proliferation of wild-type bm12.K^d^.IE CD4 T cells in B6 hosts was assessed similarly, by analysis of CSFE-staining 3 days after transfer of 5 × 10^6^ cells, with the caveat that in contrast to transfer of a monoclonal population, analysis was restricted to the relatively small (∼5%) alloreactive population nested within a large wild-type repertoire that did not undergo proliferation. Proliferation was quantified using FlowJo (Tree Star).

### Histology, Immunohistochemistry, and Immunofluorescence

Formalin-fixed hearts were paraffin mounted and stained using H&E and Weigert’s Elastin van Gieson method to delineate the internal elastic lamina and the severity of allograft vasculopathy assessed morphometrically, as reported previously (Motallebzadeh et al., 2012). Complement C4d deposition was assessed on 7-μm cryostat sections of donor heart allografts explanted after 50 days by an avidin-biotin-peroxidase technique (Vector Laboratories), using unconjugated rat anti-mouse C4 mAb (16D2; Abcam), as described previously (Win et al., 2009). GCs were quantified on 7-μm cryostat sections of recipient spleens harvested 50 days following transplant by immunofluorescence staining of B220^+^ B cells using rat anti-mouse B220 (clone RA3-6B2; BD Pharmingen) detected with Cy3-conjugated goat anti-rat IgG (clone 112-165-143, Jackson ImmunoResearch Laboratories) and peanut agglutinin (PNA)^+^ GC B cells using FITC-conjugated PNA (Vector Laboratories), as described previously (Conlon et al., 2012b). Numbers of PNA^+^ GC were expressed as a percentage of total (B220^+^) lymphoid follicles.

### In Vivo Depletion and Transfer of Donor and Recipient Lymphocyte Subsets

Donor mice were injected i.p. with 2 × 1.0 mg doses of depleting anti-CD4 mAb (YTS 191.1; hybridoma from the European Collection of Animal Cell Cultures) 6 days and 1 day before heart graft procurement. Depletion of CD4 T cells (typically >99%) was confirmed by flow cytometric analysis of peripheral blood. To confirm cardiac parenchymal CD4 T cell depletion, donor hearts were homogenized following incubation with collagenase digestion buffer, as described previously (Sivaganesh et al., 2013), with a single-cell suspension prepared by filtration through a 40-μm nylon cell strainer. CD4 T cells were quantified by flow cytometry, with a mean of 5,137 CD4 T cells identified in an untreated donor heart.

In certain experiments, recipients of CD4 T cell-depleted allografts were adoptively transferred i.v. 1 × 10^7^ donor CD4 T cells (purified with anti-mouse CD4 MicroBeads (Mitenyi) using an autoMACS Separator (Mitenyi).

Depletion of the mature recipient B cell population was achieved by injecting i.p. 250 μg depleting anti-CD20 mAb (18B12; gifted by Cherie Butts at Biogen Idec) 7 days before and 14 days after transplantation (Ueki et al., 2011). Depletion of B cells was confirmed by flow cytometry of peripheral blood mononuclear cells (PBMCs) the day before heart transplantation.

Depletion of the recipient NK cell population was achieved by injecting i.p. 500 μg depleting anti-NK1.1 (PK136; hybridoma from the European Collection of Animal Cell Cultures) 2 days and 1 day before transplant or cell transfer and three times weekly thereafter. Depletion of NK cells was confirmed by flow cytometry of PBMCs the day before transplantation or transfer.

Adoptive transfer studies of purified B6, BALB/c, bm12.K^d^, and bm12.K^d^.IE CD4 T cells into B6, *Tcrbd*^−/−^, and *Tcrbd*^−/−^.bm12 mice were performed by injecting i.v. 1 × 10^7^ cells purified as earlier.

### Statistics

Mann-Whitney U test was used for analysis of nonparametric data. Two-way ANOVA was employed for comparison of intensity of HEp-2 fluorescence scores and anti-H-2K^d^ antibody levels. Graft survival was depicted using Kaplan-Meier analysis, and groups were compared by log rank (Mantel-Cox) testing. Analysis was conducted using GraphPad 4 (GraphPad Software). Values of p < 0.05 were considered significant.

## Author Contributions

I.G.H., J.M.A., J.A.B., M.R.C., T.M.C., and G.J.P. designed the experiments. I.G.H., J.M.A., S.J.F.H., E.W., J.A., M.C.N., M.S.Q., R.M.-Z., and K.S.-P. conducted the experiments. M.C.N. designed, developed, and produced essential reagents. I.G.H., J.M.A., and G.J.P. wrote the first draft of the paper. I.G.H., J.M.A., S.J.F.H., E.W., J.A., M.C.N., M.S.Q., R.M.-Z., K.S.-P., E.M.B., J.A.B., M.R.C., T.M.C., and G.J.P. reviewed and edited the manuscript, approving the final version.

## Supplemental Information

Document S1. Supplemental Experimental Procedures and Figures S1–S6

Document S2. Article plus Supplemental Information
