# Supplementary material for: Augmentation of Recipient Adaptive Alloimmunity by Donor Passenger Lymphocytes within the Transplant
Source: Cell Rep. 2016 Apr 28;15(6):1214–27. doi: 10.1016/j.celrep.2016.04.009 (PMC4870521; doi:10.1016/j.celrep.2016.04.009)
Supplement: Document S1. Supplemental Experimental Procedures and Figures S1–S6 [file mmc1.pdf]

Cell Reports, Volume *15*

Supplemental Information

Augmentation of Recipient Adaptive Alloimmunity by Donor Passenger Lymphocytes within the Transplant

Ines G. Harper, Jason M. Ali, Simon J.F. Harper, Elizabeth Wlodek, Jawaher Alsughayyir, Margaret C. Negus, M. Saeed Qureshi, Reza Motalleb-Zadeh, Kourosh Saeb-Parsy, Eleanor M. Bolton, J. Andrew Bradley, Menna R. Clatworthy, Thomas M. Conlon, and Gavin J. Pettigrew

Supplementary methods

Cell isolation from human tissues

Human tissue (2-4g) was obtained from organ donors in whom consent for research was present. Ethical approval was granted by the local ethics committee (REC12/EE/0446) and (REC15/EE/0152) and the studies were also approved by NHS Blood and Transplant (NHSBT). Kidney, liver, spleen and heart tissue was minced, dissociated using a Gentle-MACS machine (Miltenyi Biotech, UK) then passed sequentially through 100 µm, 50 µm and 30 µm cell strainers. Addition of 44% percoll followed by centrifugation was used to remove debris.

Cells were blocked with human FcR block (Miltenyi Biotech, Bisley, UK) or normal mouse serum and incubated with antibodies (see appendix 1) for 1 hour at 4°C, followed by live/dead cell staining (Live/Dead Aqua 405, Invitrogen, Paisley, UK) for 20 minutes at room temperature. Cell surface receptor staining was undertaken at room temperature. Samples were processed on a Fortessa flow cytometer (Becton Dickinson, Basel, Switzerland) and data analysed using Flowjo software (Treestar, Ashland, TN). Antibodies were used at 1 in 200 as follows: mouse anti-human CD45 (PE, clone 2D1, eBioscience, San Diego CA), mouse anti-human CD4 (FITC, clone RPA-T4, eBioscience, San Diego CA), mouse anti-human CD8a (eFluor 450, clone SK1, eBioscience, San Diego CA), rat anti-human CCR7(PeCy7, clone 3D12, BD Pharmingen, San Diego CA), mouse anti-human CD45RA (APC, clone HI100, eBioscience, San Diego CA).

Supplementary Figures

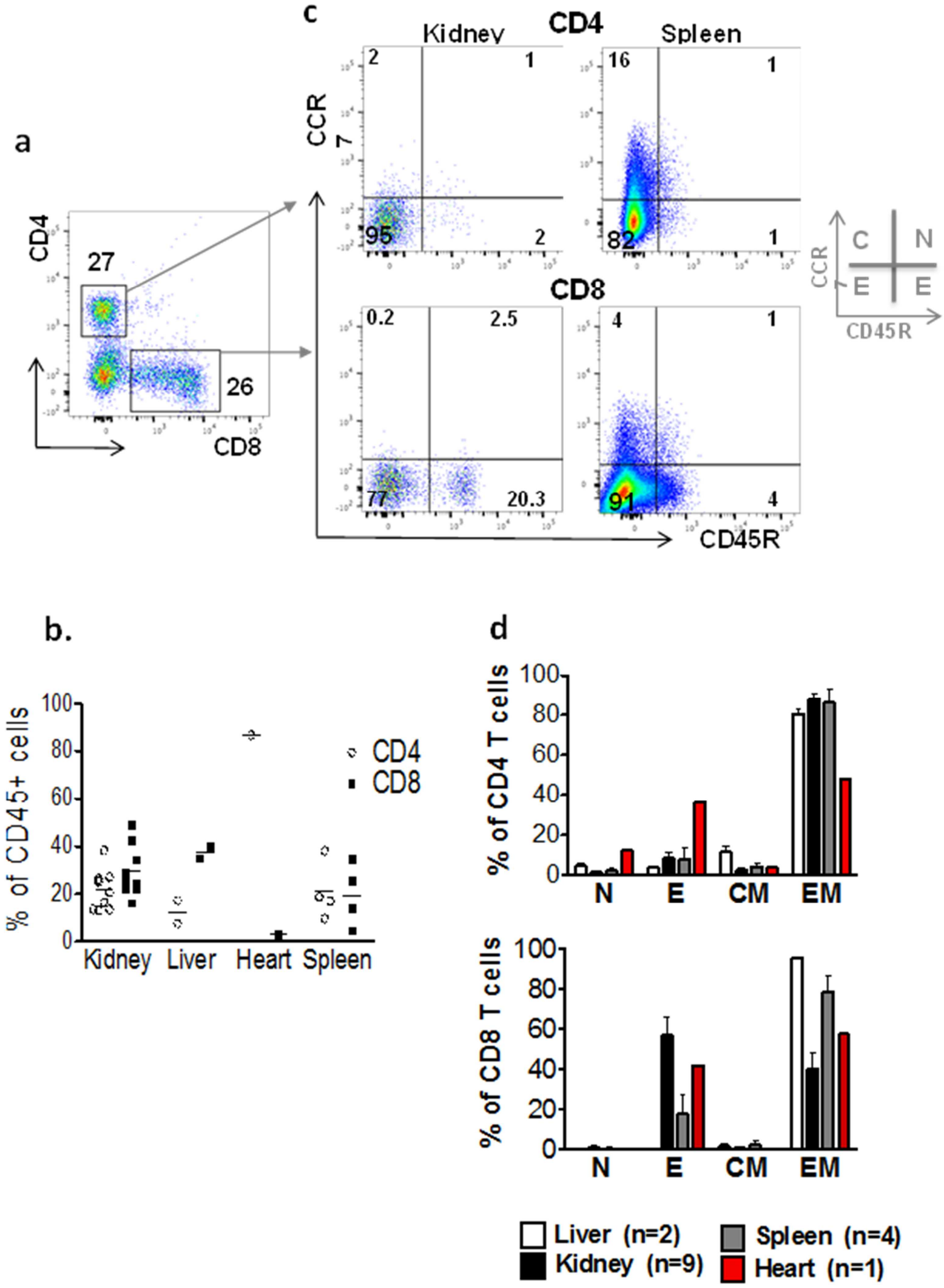

Supplementary Figure 1. Effector memory CD4 and CD8 T cells are contained within human organ transplants, related to Figure 1

(a) Flow cytometric analysis of human kidney leucocytes, gating on live/CD45+/singlets. CD4 and CD8+ populations identified. **(b)** %CD4 (open circles) and %CD8 (closed circles) T cells within CD45+ leucocyte populations isolated from human kidney, liver, heart and spleen. **(c)** representative facs plots of CD4 (upper panel) and CD8 (lower panel) T cells in human kidney and spleen stained with CCR7 and CD45RA to identify naïve (N), effector (E), central memory (CM) and effector memory (EM) – as illustrated in the schematic. **(d)** % CD4 (upper panel) and CD8 (lower panel) that are N, E, CM and EM in liver (white bars), kidney (black bars), spleen (grey bars) and heart (red bars).

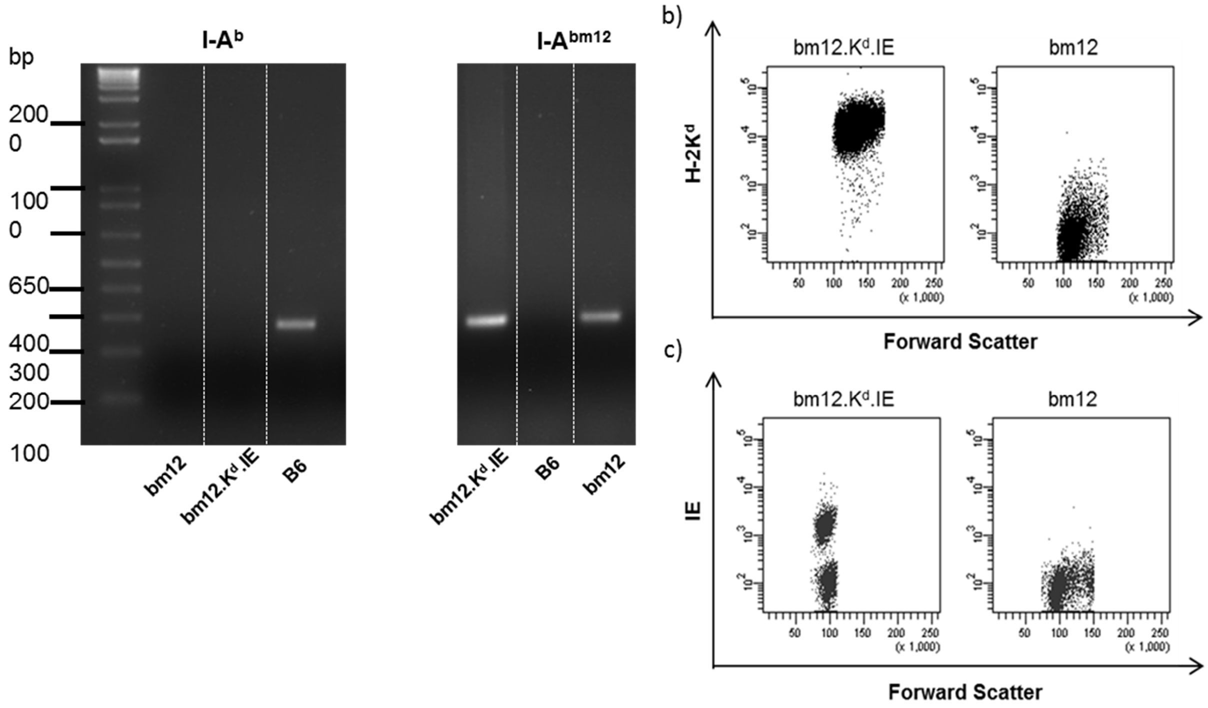

Supplementary Figure 2. Characterisation of bm12.K^d.IE donor strain, related to Figure 1.

A series of intercrosses were performed between bm12, B6.K^d and B6.IE strains to derive the bm12.K^d.IE strain (H-2^b, K^{d}K^b, A^{bm12}, E, D^b). **(a)** Flow cytometric demonstration of additional expression of classical MHC class I K^d (left panels) and class II I-E antigens (right panels) on live PBLs from bm12.K^d.IE strain; these are not expressed on parent bm12 strain. **(b)** PCR genotyping of I-A loci of bm12, B6 and bm12.K^d.IE strains using different primer pairs specific for β chain of the I-A alpha-helix region. Of note, the bm12.K^d.IE strain expresses the I-A^{bm12}, but not the I-A^b, antigen.

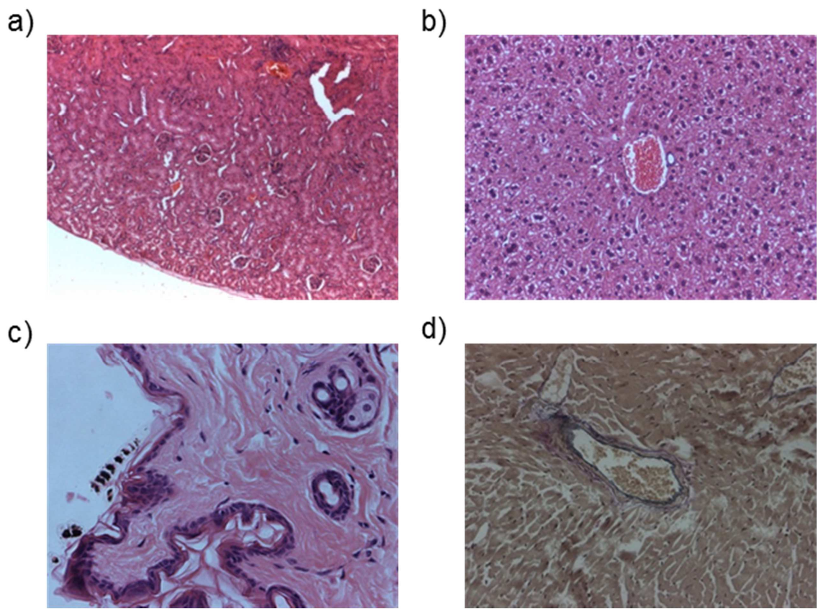

Supplementary Figure 3. The development of humoral autoimmunity in B6 recipients of bm12.Kd.IE heart allografts is not associated with autoimmune disease manifestations, related to Figure 2.

Kidney **(a)**, liver **(b)**, skin **(c)** and native heart **(d)** were retrieved from B6 recipients of bm12.Kd.IE heart allografts 100 days after transplantation and either haematoxylin and eosin staining **(a-c)** or Elastin Van Gieson's staining **(d)** of paraffin sections assessed for presence of inflammation. All tissues sampled were unremarkable and free from inflammation. Representative photomicrographs taken at different magnifications **(a)** 6.4x, **(b)** 16x, **(c,d)** 32x.

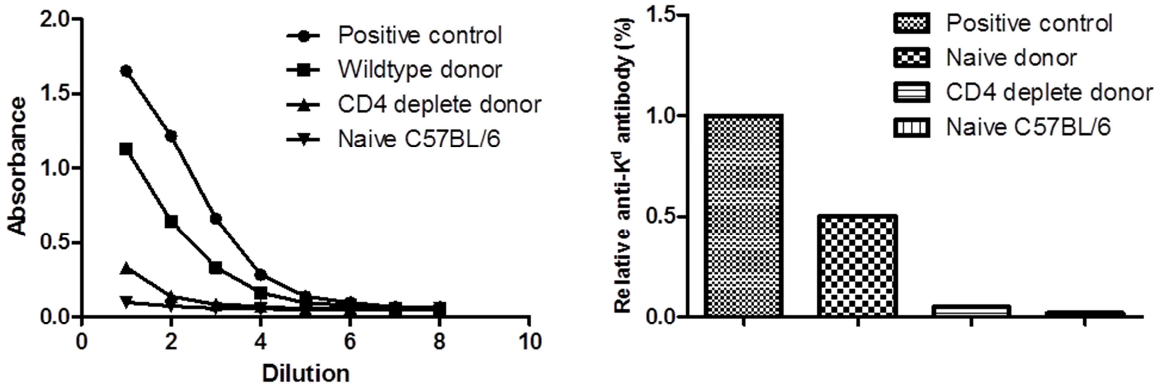

Supplementary Figure 4. Quantification of alloantibody levels, related to Figure 3

Typical absorbance versus dilution curves obtained from anti-H-2K^d IgG ELISA assay of serum samples (from C57BL/6 recipients of bm12.Kd.IE cardiac allografts with or without donor CD4 depletion 5 weeks following transplantation) (left) were subjected to area under the curve analysis and antibody levels compared to value obtained for control hyperimmune serum (positive control) (right histogram).

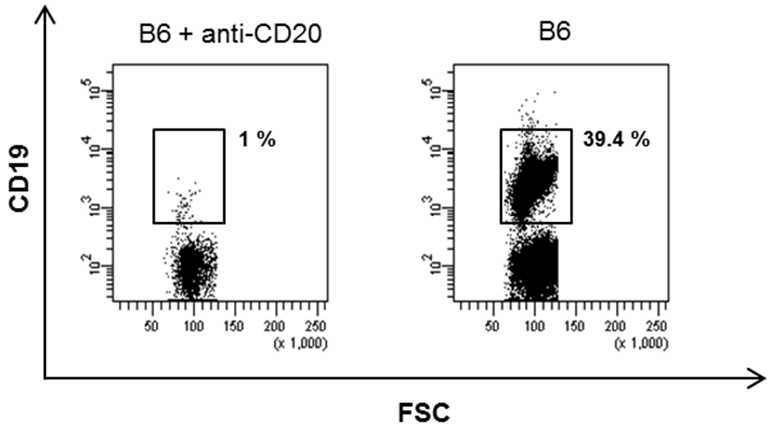

Supplementary Figure 5. Effective B cell depletion is achieved with administration of murine anti-CD20 monoclonal antibody, related to Figure 4

Treatment of donor bm12.K^d.IE mice with murine anti-CD20 mAb resulted in profound depletion of B cells in the circulation by the time of procurement of the heart graft

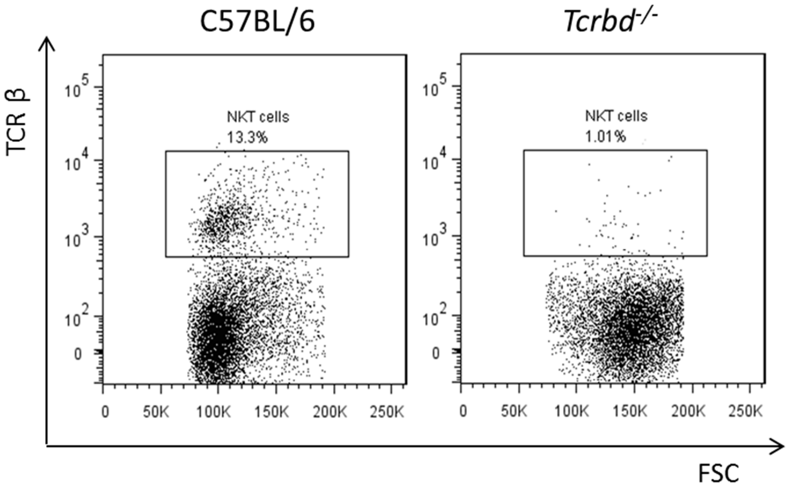

Supplementary Figure 6. ***Tcrbd$^{-/-}$*** **mice lack NKT cells, related to Figure 6**

Whereas wildtype C57BL/6 mice possess a population of NKT cells (NK1.1^{+} TCRβ^{+}), this is absent in *Tcrbd$^{-/-}$* mice.
